# Integrating biogeography, threat and evolutionary data to explore extinction crisis in the taxonomic group of cycads

**DOI:** 10.1002/ece3.2660

**Published:** 2017-03-21

**Authors:** Kowiyou Yessoufou, Barnabas H. Daru, Respinah Tafirei, Hosam O. Elansary, Isaac Rampedi

**Affiliations:** ^1^ Department of GeographyEnvironmental Management and Energy StudiesUniversity of JohannesburgJohannesburgSouth Africa; ^2^ Department of Organismic and Evolutionary Biology and Harvard University HerbariaHarvard UniversityCambridgeMAUSA; ^3^ Department of Plant SciencesUniversity of PretoriaPretoriaSouth Africa; ^4^ Department of FloricultureOrnamental Horticulture and Garden DesignAlexandria UniversityAlexandriaEgypt

**Keywords:** Biogeography, cycad tree of life, diversity hotspots, evolutionary distinctiveness, extinction risk, gymnosperms

## Abstract

Will the ongoing extinction crisis cause a severe loss of evolutionary information accumulated over millions of years on the tree of life? This question has been largely explored, particularly for vertebrates and angiosperms. However, no equivalent effort has been devoted to gymnosperms. Here, we address this question focusing on cycads, the gymnosperm group exhibiting the highest proportion of threatened species in the plant kingdom. We assembled the first complete phylogeny of cycads and assessed how species loss under three scenarios would impact the cycad tree of life. These scenarios are as follows: (1) All top 50% of evolutionarily distinct (ED) species are lost; (2) all threatened species are lost; and (3) only all threatened species in each IUCN category are lost. Finally, we analyzed the biogeographical pattern of cycad diversity hotspots and tested for gaps in the current global conservation network. First, we showed that threatened species are not significantly clustered on the cycad tree of life. Second, we showed that the loss of all vulnerable or endangered species does not depart significantly from random loss. In contrast, the loss of all top 50% ED, all threatened or all critically endangered species, would result in a greater loss of PD (Phylogenetic Diversity) than expected. To inform conservation decisions, we defined five hotpots of diversity, and depending on the diversity metric used, these hotspots are located in Southern Africa, Australia, Indo‐Pacific, and Mexico and all are found within protected areas. We conclude that the phylogenetic diversity accumulated over millions of years in the cycad tree of life would not survive the current extinction crisis. As such, prioritizing efforts based on ED and concentrating efforts on critically endangered species particularly in southern Africa, Australia, Indo‐Pacific, and Mexico are required to safeguarding the evolutionary diversity in the cycad tree of life.

## 1 Introduction

Originated ~300 million years ago (Hendricks, 1987), cycads are a fascinating plant group sharing morphological characteristics of ferns and angiosperms (Brenner, Stevenson, & Twigg, 2003; Norstog & Nicholls, 1997). They had once a worldwide distribution particularly in the Mesozoic era (Hermsen, Taylor, & Taylor, 2009), but the extant cycads, which diverge around 12–2 Ma (Nagalingum et al., 2011), are restricted to tropical and subtropical regions of the world. Almost 70% of cycad species are threatened with a high risk of extinction (IUCN, 2010; Osborne, Calonje, Hill, Stanberg, & Stevenson, 2012). As the susceptibility of species to extinction is linked to their past evolutionary history (Davies et al., 2011; Vamosi & Wilson, 2008), reconstructing the tree of life of a particular taxonomic group is likely to inform, not only our understanding of the pattern of extinction risk in the group (Davies et al., 2011; Purvis, Agapow, Gittleman, & Mace, 2000), but also how the tree of life could be pruned by species loss (Davies & Yessoufou, 2013; Mooers, Gascuel, Stadler, Li, & Steel, 2012; Parhar & Mooers, 2011; Purvis et al., 2000). Such understanding would, in turn, guide actions toward the preservation of the evolutionary diversity accumulated in the tree of life (Faith, 1992; Forest et al., 2007). For example, a strong phylogenetic signal in a threat can guide the prediction of the threat status of a particular species that has not yet been assessed.

In their study, Nee and May (1997) demonstrated that “80% of the underlying tree of life can survive even when approximately 95% of species are lost.” This finding has been criticized on the ground that it is based on the most unrealistic tree topology, that is, a coalescent‐type model of tree shape (Davies, 2015; Davies & Yessoufou, 2013; Mooers et al., 2012), a model known to be “tip heavy” with most terminal branches short and clustered toward the present, as opposed to the more common and realistic Yule and the birth–death models (Davies, 2015; Davies & Yessoufou, 2013; Mooers et al., 2012; Yessoufou & Davies, 2016). Based on more realistic models of tree shape, the loss of evolutionary history with the loss of species can be rapid (Mooers et al., 2012), and this can be further amplified by nonrandom extinction (Davies & Yessoufou, 2013; Heard & Mooers, 2000; Purvis et al., 2000; but see also Parhar and Mooers, 2011). Because the phylogeny of cycads, assembled at many occasions (Condamine, Nagalingum, Marshall, & Morlon, 2015; Nagalingum et al., 2011; Yessoufou, Bamigboye, Daru, & van der Bank, 2014), is “remarkable for its long branches subtending the late Cenozoic radiations” (Nagalingum et al., 2011), an unusual topology matching a coalescent model of evolution (root is very old, but the crown clades are very young), the phylogenetic pattern of extinction risk and the way this extinction would prune the cycad tree of life might be different from the common patterns reported for most taxonomic groups.

To minimize the impact of species extinction on the tree of life and prevent the loss of species playing unique roles in the ecosystems, traditional conservation efforts prioritize endemic, rare, or threatened species or species‐rich regions (Selig et al., 2014; Venter et al., 2014), and high‐altitude habitats (e.g., mountains) are also given priority for the preservation of ancient lineages (Fjeldså, Bowie, & Rahbek, 2012; Fjeldså & Lovett, 1997). These studies, however, do not explicitly address the preservation of the evolutionary components of biodiversity, although evidence has now shown that either of these traditional metrics (e.g., species richness, endemism, rarity, or biodiversity hotspots) is not a silver bullet conservation strategy (Daru, Van der Bank, & Davies, 2015; Mouillot et al., 2016; Orme et al., 2005). Consequently, recent studies call for an integrative approach (Daru et al., 2015; Jetz et al., 2014; Mazel, 2014; Tucker, Cadotte, Davies, & Rebelo, 2012), aiming to preserve diverse facets of biodiversity that includes necessarily the evolutionary component which is thought to contribute significantly to ensuring a sustainable ecosystem functioning in the face of global change (Cadotte, 2013; Cadotte, Dinnage, & Tilman, 2012; Faith et al., 2010; Forest et al., 2007). From this perspective, the traditional species richness metric (SR) is increasingly analyzed alongside many other facets of diversity (see, e.g., Daru & le Roux, 2016; Daru et al., 2015), including phylogenetic diversity (PD; Faith, 1992), phylogenetic endemism (PE; Rosauer, Laffan, Crisp, Donnellan, & Cook, 2009), evolutionary distinctiveness (ED; Isaac, Turvey, Collen, Waterman, & Baillie, 2007), the combination of ED with species global endangerment (EDGE; Redding & Mooers, 2006), and corrected weighted endemism (CWE) (see details of these metrics below in Materials and methods). The use of ED, in particular, to inform conservation actions has recently been shown to be efficient in capturing most evolutionary history accumulated in a particular tree of life (Redding, Mooers, Şekercioğlu, & Collen, 2015; Redding et al., 2008), especially when integrated within a biogeographical framework (Jetz et al., 2014). ED provides a number of advantages in conservation. Firstly, ED, used to guide prioritization efforts at a global scale, can capture simultaneously species that need urgent attention at local scale (Redding et al., 2015). Secondly, ED can also capture broadly the biology of a particular group (Redding, DeWolff, & Mooers, 2010), and lastly, the preservation of high‐ED species may lead to the preservation of uniquely divergent genomes (Warren et al., 2008). All these studies that address explicitly the preservation of the evolutionary diversity or employ an integrative approach to inform conservation, however, focus on vertebrates (Jetz et al., 2014; Redding et al., 2010) and increasingly on angiosperms (Daru & le Roux, 2016; Daru et al., 2015; Vamosi & Wilson, 2008) with no equivalent efforts ever made on gymnosperms, although the latter has a unique evolutionary history in plant kingdom (Nagalingum et al., 2011).

Here, we use a biogeographical framework to tackle this critical knowledge gap for the iconic but threatened cycads, a gymnosperm group at the brink of extinction (I.U.C.N., 2010). First, we assemble the first ever complete phylogeny of cycad species. Second, we use this phylogeny to (1) revisit briefly the evolutionary history of cycad species diversification, (2) analyze the phylogenetic pattern of extinction risk in cycad group, and (3) investigate how this extinction might prune the cycad tree of life. Third, we contrast hotspots of multiple diversity facets of cycads across biogeographical regions and assess how well the current global protected area network preserves cycad diversity hotspots and cycad species that need urgent attention.

## 2 Materials and Methods

### 2.1 Assembling a complete list of cycad species

The world list of cycads has changed several times owing to the high morphological similarities among species (morphological stasis), resulting in a long list of synonyms (Osborne et al., 2012). In their recent work, Osborne et al. (2012) summarized the existing knowledge of morphology and ecology of cycads, on which they based their taxonomic discrimination to distinguish 331 cycad species globally, thus providing the most recent world list of cycad species. Earlier, Nagalingum et al. (2011) used DNA data to assemble a comprehensive phylogeny of global cycads that include 199 species. In this study, Osborne et al.'s list is used as the reference list, while also taking some nuances into account based on Nagalingum et al.'s DNA‐based phylogeny. Specifically, based on their positions on the phylogeny, Nagalingum et al. (2011) distinguished *Cycas media ensata* and *C. media*,* C. pectinata* A and *C. pectinata* B as well as *Zamia furfuracea* A and *Z. furfuracea* B. Also, *Z. picta*,* Z. lawsoniana*,* Z. kickxii*, and *Z. amblyphyllidia* are all maintained in Nagalingum et al. (2011) as distinct species, whereas Osborne et al. (2012) considered them as synonyms of *Z. variegata*,* Z. loddigesii*,* Z. pygmaea*, and *Z. erosa*, respectively, based on their morphological features. Finally, following Lindström (2009), Osborne et al. (2012) did not recognize the genus *Chigua*, while this genus was maintained in Nagalingum et al. (2011). In this study, Nagalingum et al.'s DNA‐based nuances are taken into account and combined with Osborne et al.'s species delimitation to distinguish 339 taxa of cycads. These taxa are presented in Table S1 along with their global distribution.

### 2.2 Assembling a complete phylogeny of cycad species

To assemble a complete phylogeny of cycads, the recently proposed approach of Thomas et al. (2013) that assembles a complete phylogeny with soft taxonomic inferences was used. This approach requires (1) a DNA‐based phylogeny to be used as a constraint tree and (2) the taxonomic information of species missing in the constraint tree. Following Thomas et al. (2013), three types of species were defined: type 1 (comprising species for which DNA sequences are available), type 2 (species with no DNA sequence but are congeners of type 1 species), and type 3 (species that have no DNA data and have no congeners among type 1 species). In this study, type 1 species comprises 199 species (see details below) and there is no type 3 species. Thomas et al.'s approach to integrate type 1 and type 2 species relies on two assumptions: Taxonomic groups (e.g., genera) are monophyletic, and there is a reasonable edge length and topology priors. These two assumptions are met for cycads as all cycad genera are monophyletic and a DNA‐based dated phylogeny, used as constraint tree, does exist (see Nagalingum et al., 2011).

The constraint tree was assembled using the DNA sequences of the nuclear region PHYP for 199 species (type 1 species) of all 339 cycad taxa. The matrix of PHYP DNA sequences, retrieved from TreeBASE (www.treebase.org; #11891; Nagalingum et al., 2011), includes a proportional sampling within the large cycad genera (see Nagalingum et al., 2011 for details) and comprises all the 11 currently defined genera. An XML file was generated in the program BEAUTi, which was used to reconstruct a dated phylogeny based on a Bayesian MCMC approach implemented in the BEAST program. In the process of dated tree reconstruction, GTR + I + Γ was selected as the best model of sequence evolution based on the Akaike information criterion evaluated using MODELTEST (Nylander, 2004). Also, a Yule process was selected as the tree prior, with an uncorrelated relaxed lognormal model for rate variation among branches. Further, a normal prior distribution and several secondary calibration points were applied: *Encephalartos* crown node (11.3648 Myr), *Macrozamia* crown node (7.4836 Myr), *Lepidozamia* crown node (7.914 Myr), *Cycas* crown node (12.7977 Myr), *Zamia* crown node (11.2534 Myr), *Dioon* crown node (12.1254 Myr), *Encephalartos*–*Lepidozamia* (39.7442 Myr), and (*Encephalartos*–*Lepidozamia*)–*Macrozamia* (49.037 Myr) (Nagalingum et al., 2011). Monte Carlo Markov chains were run for 100 million generations with trees sampled every 10,000 generations. Log files, including prior and likelihood values, as well as the effective sample size (ESS), were examined using TRACER (Rambaut & Drummond, 2007). ESS values varied between 1,058 and 7,826 for the age estimates, confirming stationarity. Of the resulting 10,001 trees, the first 2,500 trees were removed as burn‐in and the remaining trees were combined using TREEANNOTATOR (Rambaut & Drummond, 2007) to generate a maximum clade credibility (MCC) tree. The following species were used as outgroups: *Ginkgo biloba*,* Cryptomeria japonica, Araucaria heterophylla, Pinus strobes, Pseudotsuga menziesii, and Abies firma* (Nagalingum et al., 2011).

To integrate the 140 type 2 species into the constraint tree, a simple taxon definition file that lists all species (types 1 and 2) along with clade names (genus names) was formed. Then, the R library PASTIS (Thomas et al., 2013) was used to integrate type 2 species into the constraint tree as explained in Thomas et al. (2013); this results in a MrBayes input file that is executed in MrBayes 3.2 (Ronquist & Huelsenbeck, 2003) to generate a complete phylogeny of cycads that combines genetic data (type 1) and taxonomic data (type 2). This approach has recently been used to assemble a complete phylogeny for birds (Jetz, Thomas, Joy, Hartmann, & Mooers, 2012; Jetz et al., 2014).

### 2.3 Evolutionary diversification history of cycads

Here, we intend to (1) describe the best model of evolution that matches the temporal pattern of cycad species accumulation and (2) test for rate heterogeneity across lineages to identify the clade, if any, that might experience an unusual rate of species accumulation in the group. Six models were tested, including two rate constant models (pure speciation and birth–death models) and four rate variable models, that is, the density‐dependent exponential (DDX) model, the density‐dependent linear (DDL) model, the Yule2rate model, and the Yule3rate model. We fitted these models under the maximum‐likelihood criterion and selected the best model based on ΔAIC_RC_ = AIC_H0_ − AIC_H1_, where AIC_H0_ is the AIC score of the best rate constant model and AIC_H1_ is the AIC score of the best rate variable model. This model comparison was performed using the function fitdAICrc in the R package *laser* (Rabosky 2007). ΔAIC_RC_ > 0 indicates that the best of the rate variable models is also the best model for the observed diversification pattern, whereas ΔAIC_RC_ < 0 favors the best rate constant model as the best model for overall diversification (Rabosky & Lovette, 2008).

The test for rate heterogeneity across lineages was conducted using two methods, the Δ_1_ statistic test (Moore, Chan, & Donoghue, 2004) and the PRC (parametric rate comparison) method (Shah, Fitzpatrick, & Fordyce, 2013). Moore et al.'s test is based on the whole tree topology to detect nodes associated with significant shifts in diversification rate. This test was performed using the R package *apTreeshape* (Bortolussi, Durand, Blum, & François, 2006). The PRC method identifies subclades of a tree that have relatively higher rates of diversification, by explicitly assessing the distribution of branch lengths rather than the distribution of cladogenic events across the entire tree. The PRC analysis was performed using the R package *iterates* (Fordyce, Shah, & Fitzpatrick, 2014).

### 2.4 Phylogenetic analysis of extinction risk in cycad lineage

Prior to analysis, threat status for each species was retrieved from IUCN database, and when information is unavailable on IUCN, threat status was compiled from Osborne et al. (2012). Then, species were categorized as threatened (when they belong to one of the IUCN categories VU, EN, and CR) and nonthreatened (categories LC and NT). The phylogenetic signal of threat status (threatened vs. nonthreatened) was tested using Fritz and Purvis (2010) D statistic (R package *Caper*; Orme et al., 2012). D < 0 when there is a strong phylogenetic signal, while D > 1 is indicative of a phylogenetic overdispersion. The significance of D values was tested by comparing the observed D value to 0 (expected value for a phylogenetically conserved threat status under a Brownian motion model) and 1 (random expectation). The *p* values for significance tests are reported as *P*_BM_ (giving the result of testing whether D is significantly different from 0) and *P*_rand_ (giving the result of testing whether D is significantly different from 1).

Using one‐way ANOVA, we further tested whether evolutionary isolated species were more threatened comparing the evolutionary age of threatened vs. nonthreatened cycad species. Species evolutionary age was approximated using ED values (see also Jetz et al., 2014). In addition, we tested the relationships between threat levels (LC, NT, VU, EN, and CR) and ED values using ANOVA.

### 2.5 Extinction risk and the future of cycad tree of life

We assessed how the current extinction crisis in cycads might impact the evolutionary history (PD) accumulated on the cycad tree of life. This assessment was performed in three ways. On the one hand, we assessed whether the loss of the top 50% ED species (165 species) would cause a severe loss of PD more than expected at random (after 165 species were randomly pruned 100 times from the CToL). On the other hand, we assessed whether the loss of all currently threatened species (215 species) would lead to the loss of more PD than expected under the scenario where 215 species were randomly pruned from the CToL (after 100 randomizations). Finally, because losing 215 species out of 339 species may have very little power to demonstrate a greater‐than‐random loss of PD, we further tested how the loss of all threatened species in each IUCN categories [VU (78 species), EN (70 species), and CR (67 species)] would impact the CToL.

### 2.6 Distribution data and hotspots of cycad diversity

We retrieved from GBIF (www.gbif.org, June 2015) all geographical data available on cycad, particularly the GPS coordinates. These data were cleaned as follows. First, we established our list of all cycad as explained above. Then, we used the “The World List of Cycads” (Osborne et al., 2012) to have the list of all synonyms of cycad species. Next, we removed from our GBIF data all species that fall in the ocean. Based on synonyms, we also removed all duplicates in our data. Finally, we used very well‐known geographical distribution of cycads (Osborne et al., 2012) to remove all false species occurrence; for example, in our GBIF data, some *Encephalartos spp*. are found in the USA while we obviously know that all *Encephalartos* are African. The world map was then gridded at a resolution of 100 × 100 km, a resolution commonly used in global‐scale macroecological studies (Daru et al., 2016; Storch, Keil, & Jetz, 2012). In addition, species ranges (i.e., extent of occurrence) and altitudinal information (min., max., and mean altitude) were all retrieved from IUCN database (I.U.C.N., 2010).

The distributional GPS coordinates of cycads were projected onto a Behrmann equal area cylindrical projection and aggregated to 1° × 1° degree grids (ca. 110 × 110 km at the equator) to record species’ presence/absence within grid cells. We mapped hotspots for five diversity indices, SR, WE, PD, PE, and EDGE singly and cumulatively using the *merge* function in the R package *raster* (Hijmans, 2015). SR is simply the number of species per grid cell. PD is the sum of evolutionary history (phylogenetic branch lengths) accumulated in a set of species (Faith, 1992). PE, which is a range‐weighted variant of PD, identifies geographical concentrations of phylogenetically and geographically restricted species (Rosauer et al., 2009). As such, PE mirrors how much PD is captured by a clade and how much of that clade is restricted to a given geographical region. PE was measured by multiplying each phylogenetic branch length by the fraction of its range found within a given area (Rosauer et al., 2009). Species endemism was calculated using the CWE metric (corrected weighted endemism, Crisp, Laffan, Linder, & Monro, 2001), which represents endemism as the sum of the species counts for each grid cell, with each species weighted by the inverse of the number of grid cells it is found in. The EDGE metric was measured by calculating the evolutionary distinctiveness (ED) score of each species and combining this score with the global endangerment (GE) of the species as measured by IUCN conservation threat categories (Isaac et al., 2007). Prior to EDGE evaluation, data deficient species were excluded from the dataset and GE was coded as follows (Butchart et al., 2005): Least Concern = 0, Near Threatened and Conservation Dependent = 1, Vulnerable = 2, Endangered = 3, Critically Endangered = 4. EDGE scores were then calculated for all species using the standard algorithm: EDGE = ln(1 + ED) + GE * ln(2).

We quantified hotspots using the 2.5% threshold, a criterion commonly used to define biodiversity hotspots by selecting the richest 2.5% of grid cells for each metric (Ceballos & Ehrlich, 2006; Daru & le Roux, 2016; Daru et al., 2015).

Last, we tested the effectiveness of the global terrestrial network of reserves in protecting diversity hotspots of the various cycads using the World Database on Protected Areas (IUCN & UNEP‐WCMC 2015). Here, we considered a grid cell to be protected if at least 50% of its area overlaps any extent of the current protected areas. All spatial analyses were conducted in R (R Development Core Team, 2013). All the maps were generated using ArcMap 10.0 (ESRI, 2010).

## 3 Results

### 3.1 Cycad tree of life and brief evolutionary history of cycad diversification

**Figure 1:**
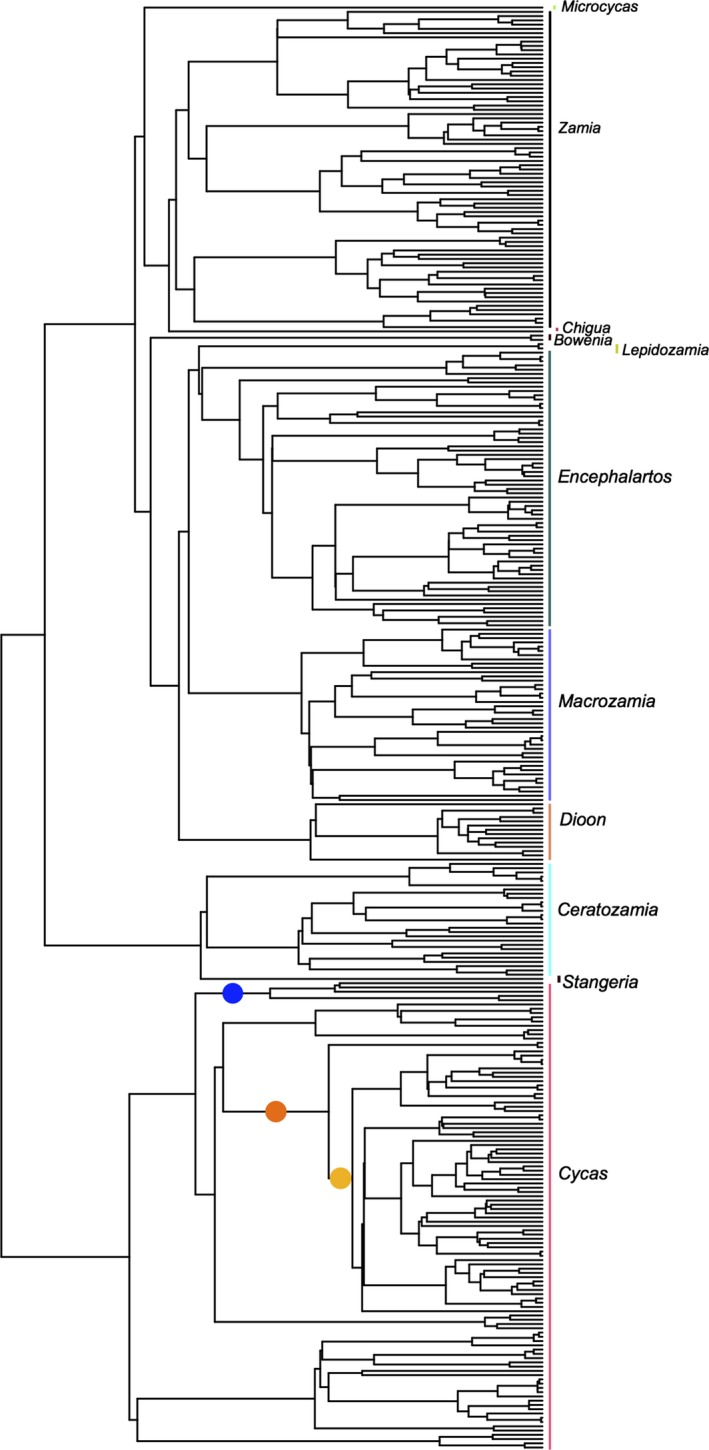
A first complete phylogeny of cycads comprising 339 taxa. Clades with higher or lower diversification rates according to the parametric rate comparison test are indicated by hot or cold colors, respectively. The outgroups were removed for the purpose of diversification rate analysis

We assembled the first complete phylogeny of cycads comprising 339 taxa, henceforth referred to as the cycad tree of life (CToL). As expected, there is no surprise in the topology of the CToL: The origin of each clade (here genus) is decoupled from its later evolutionary radiation toward the tips; each genus is monophyletic and is subtended by long branches to the origin (phylogenetic fuses), with shorter terminal branches subtending all species within each clade (Figure 1). The total evolutionary diversity accumulated in the CToL (as measured by total Faith's PD) is equal to ~ 9 billion years (sum of all ED values; see Table S1). This diversity has been accumulated through a nonconstant diversification rate matching a yule3rate diversification model (*r*_1_ = .07, *r*_2_ = .01, *r*_3_ = .04; ΔAIC_RC_ = 5.68) with the genus *Cycas* being the most rapidly diversifying clade (Figures 1 and S1).

### 3.2 Phylogenetic analysis of extinction risk in cycads

**Figure 2:**
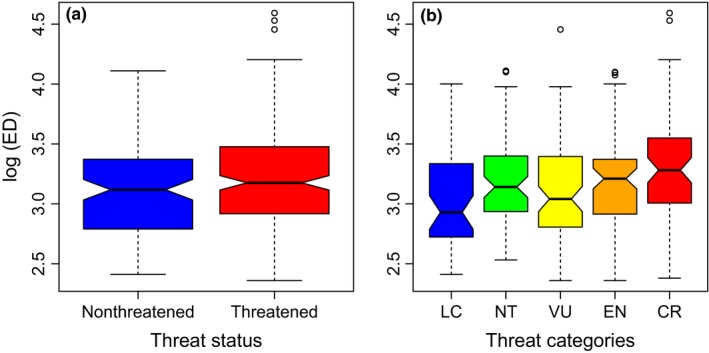
Evolutionary distinctiveness of cycad species in relation to (a) threat status and (b) IUCN threat categories

We used the CToL to assess how the evolutionary diversification history of cycad predisposes them to extinction. All threatened cycad species are only weakly clustered on the CToL (D = 0.848, *P*_rand_ = 0.007, *P*_BM_ = 0.00) and are not, on average, evolutionarily older than nonthreatened species (one‐way ANOVA, *p *=* *.228; Figure 2a). However, highly threatened species tend to be more evolutionarily distinct (one‐way ANOVA, *p *=* *.027; Figure 2b).

### 3.3 Impacts of species loss on the cycad tree of life

**Figure 3:**
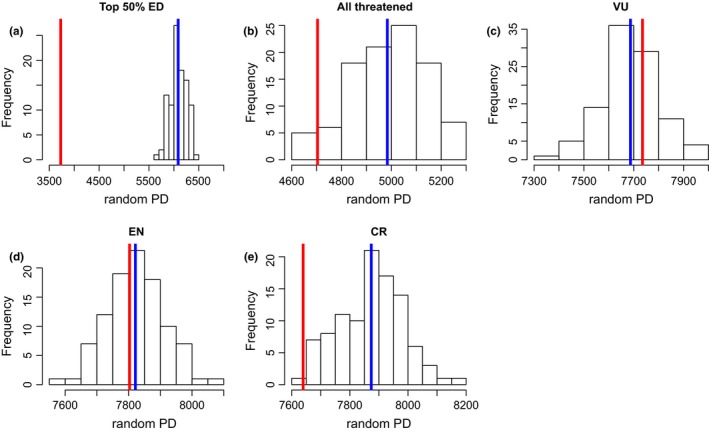
Patterns of remaining PD on the cycad tree of life under different scenarios of species loss. Red line = observed remaining PD in a scenario where (a) the top 50% ED species is lost; (b) all the 215 threatened cycad species are lost; and (c–e) all VU, EN, and CR species are lost, respectively. The histogram depicts the pattern of remaining PD on the tree after equivalent number of species in each scenario is pruned randomly from the tree. The blue line indicates the mean of PD remaining after random pruning

We found that if all threatened cycad species go extinct, we would lose ~53% of the total PD (PD_threatened_ = 4.7039 billion; PD_total_ = 8.8421 billion) accumulated in the CToL. We then tested whether the loss of the top 50% ED species (165 species) would translate in a dramatic loss of PD than expected at random. As expected, we found that the loss of the top 50% ED species would result in a greater loss of PD than expected at random (Figure 3a). We also tested whether the loss of all 215 threatened cycad species would result in a disproportionate loss of PD. Similarly, we found that we would lose more PD than that predicted (Figure 3b). We further explored the potential impact of the loss of all species in each threat category. We found that the loss of all vulnerable (VU) and endangered (EN) species is no different from random loss (Figure 3c,d). However, the loss of all critically endangered (CR) species would lead to a greater loss of PD than expected (Figure 3e).

### 3.4 Biogeography of cycad hotspots and EDGE‐informed conservation measures

**Figure 4:**
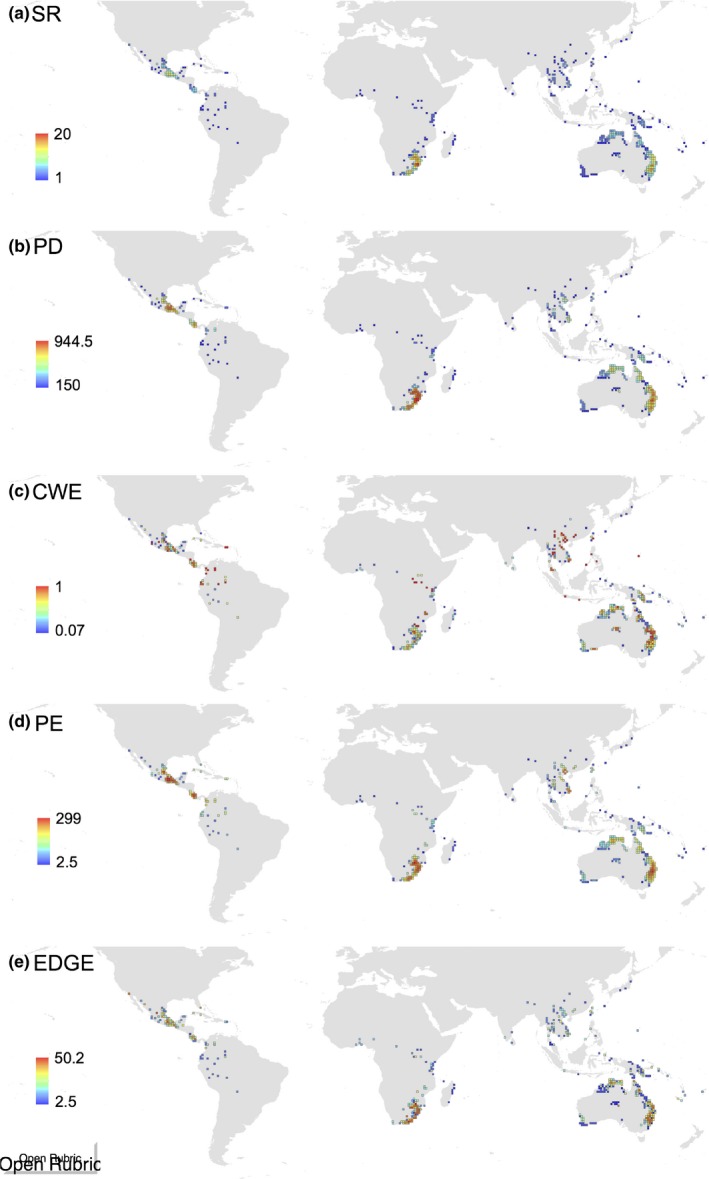
Spatial distribution of multiple cycad diversity metrics across 100 × 100 km equal area grids (Behrmann projection): (a) species richness, (b) phylogenetic diversity, (c) mean species endemism, (d) phylogenetic endemism, and (e) mean EDGE. The map was generated using ArcMap 10.0. Color scales are based on equal interval categories centered on zero and labeled with median values

Cycads have a tropical and subtropical distribution with the highest SR (max. 20 species in 100 × 100 km grid cell) in southern Africa, eastern Australia, and the Neotropics (Figure 4). Apart from southern China emerging as a high‐PD region, PD follows a geographical pattern similar to that of SR. PE and EDGE scores follow the same pattern as PD. The highest values of CWE are found in southern, eastern, and central Africa, Australia, the Neotropics, and southern China. Southern Africa, Australia, and America (Neotropics) have the highest average values of ED (Figure S2). In comparison with other taxonomic groups that receive special conservation attention, cycads score highest on the ED ranking (see Figure S3). Also, the America's cycads are the most evolutionarily distinct (*p *=* *.003; see Figure S3). There was no correlation between ED and geographical range size (*p *=* *.525) and altitude (*p *=* *.894).

**Figure 5:**
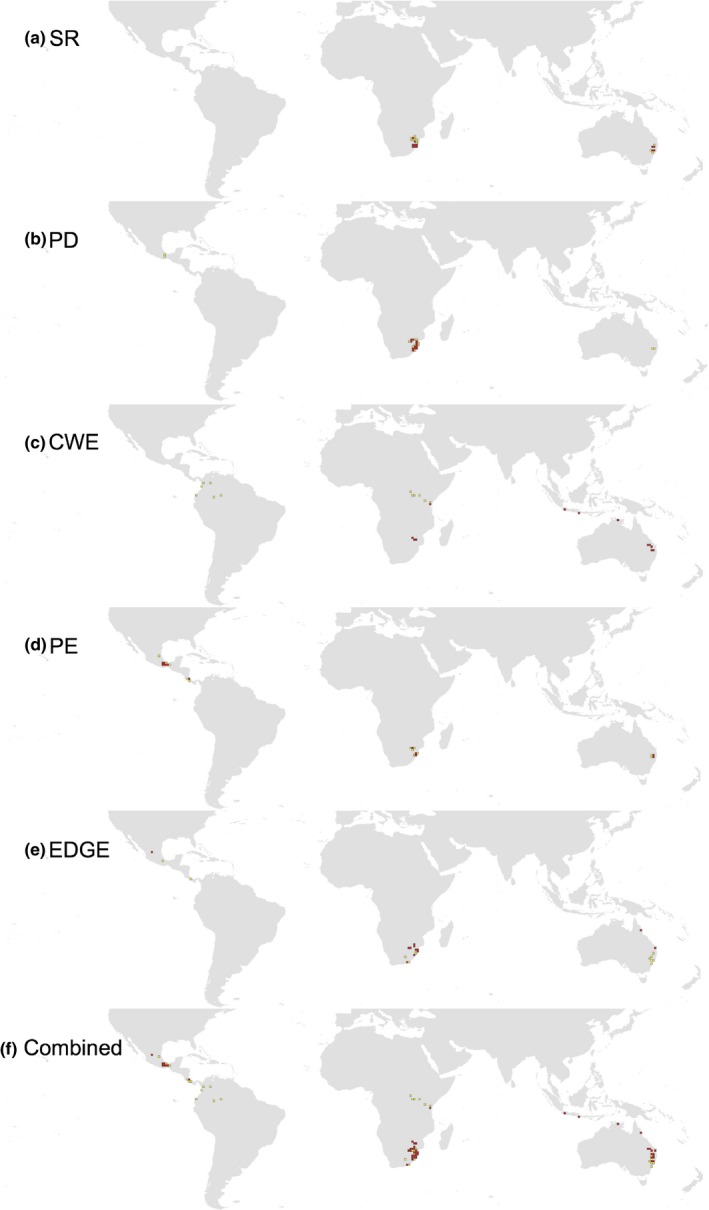
Hotspots of cycad diversity. Hotspots of (a) species richness, (b) phylogenetic diversity, (c) mean species endemism, (d) phylogenetic endemism, (e) mean EDGE, and (f) cumulative map of all five hotspots combined together. The hotspots are grid cells with the highest 2.5% of the diversity scores (shown in red), and the 5.0% hotspots are shown in yellow. The map was generated using ArcMap 10.0. Color scales are based on equal interval categories centered on zero and labeled with median values

The five hotspots of cycad diversity based on SR, PD, WE, PE, and EDGE scores defined as the 2.5% threshold are concentrated only in few regions, cumulatively occupying 582,000 km^2^, that is, only 9.9% of the terrestrial ranges of all cycads (Figure 5). Hotspots of SR are concentrated in southern Africa and eastern Australia (Figure 5a). This matches the pattern of PD hotspots, but with more cells in southern Africa and fewer in eastern Australia (Figure 5b). Hotspots of species endemism (CWE) are concentrated in few areas of southern Africa, northeast Australia, and some parts of the Indo‐Pacific (Figure 5c). The same pattern holds for PE, but with additional hotspots in Mexico (Figure 5d). The EDGE hotspots are more concentrated in southern Africa and few cells in northeast Australia (Figure 5e).

**Figure 6:**
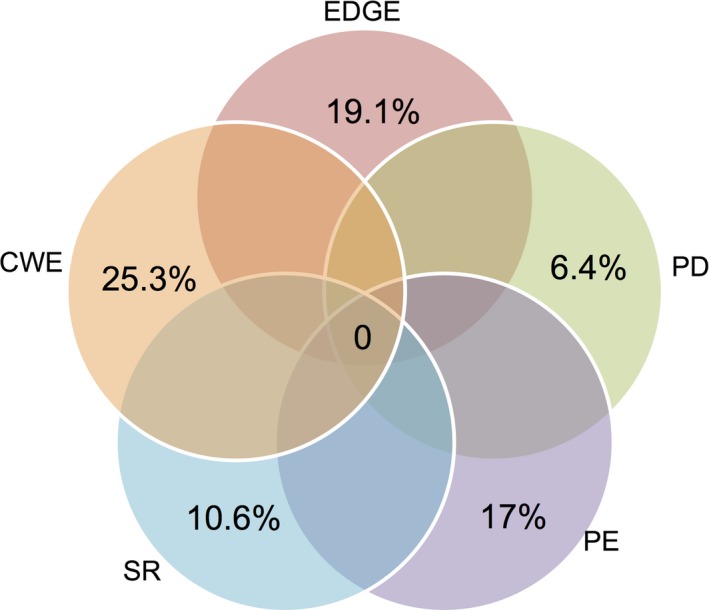
Venn diagram of spatial overlap and mismatch between hotspots of cycad diversity. SR, species richness; PD, phylogenetic diversity; CWE, corrected weighted endemism; PE, phylogenetic endemism; EDGE, evolutionary distinctiveness and global endangerment

Perhaps surprisingly, no cells are shared among all five diversity hotspots (Figure 6), and all hotspots are found within the current global network of protected areas, a finding that can be misleading with regard to species‐specific conservation measures. With this in mind, a complete ranking of cycad species based on EDGE score is provided (Table S1). EDGE scores range from 2.497 (*Cycas clivicola*) to 7.375 (*Microcycas calocoma*) (SD = ±1.06), making *M. calocoma* the top priority in conservation program. The dominant genera in the top 50 EDGE species are *Zamia* (21 species) and *Encephalartos* (10 species), followed by *Ceratozamia* (eight species) and *Cycas* (six species). From a biogeographical perspective, the cycads of the New World are dominant in the top EDGE scores with 32 species in the top 50 EDGE scores followed by the African cycads (*Encephalartos*; 10 species). Several high‐EDGE species are not found in protected areas.

## 4 Discussion

At global scale, most cycad diversification events occurred recently (Nagalingum et al., 2011), suggesting that the current geographical pattern of extant cycads was shaped by relatively recent evolutionary events. The Americas’ cycads stand out as the most evolutionarily distinct species. This implies that the Neotropics are key regions in the diversification history of cycads and therefore deserve to be prioritized when making conservation decision on a global scale. It also suggests that the synchronous radiation of all cycads at global scale (Nagalingum et al., 2011) may mask important diversification events that occurred at regional scale. For example, the particularity of the climate fluctuation in Africa during the Pliocene–Pleistocene transition has mediated the diversification and the geographical pattern of cycads in Africa (Yessoufou et al., 2014). Vicariant speciation followed by long‐distance transoceanic dispersal events has shaped cycad distribution in Australasia in the late Miocene (Xiao & Möller, 2015), whereas the Neogene tectonically driven paleogeographical events played an important role in shaping cycad diversity in the Neotropics (Rull, 2008, 2011).

Despite these region‐based diversification events, it is the most broadly distributed clade, that is, the genus *Cycas* that has the highest diversification rate. Indeed, as opposed to most cycad clades that have a restricted geographical range, *Cycas* has the widest distribution, from eastern Africa eastward to the Pacific islands and from China and southern Japan southward to Australia (Hill, 2004). This widespread distribution of *Cycas*, a genus thought to have originated in South China (Xiao & Möller, 2015), is a result of long‐distance transoceanic dispersal events that were likely facilitated by the development of a key innovation such as spongy endocarp (de Laubenfels & Adema, 1998). The fast radiation of *Cycas* that we detect is likely a result of vicariant speciation events promoted by the physical barrier of the Red River Fault between South China and Indochina blocks in the late Miocene (Xiao & Möller, 2015).

These global and regional diversification events result in the radiation of 339 cycad taxa (see Table S1) that are, unfortunately, subject not only to high risk of extinction (I.U.C.N., 2010) but also to the risk of losing an important amount of evolutionary diversity. Such risk is ill afforded in the context of multiple calls to preserve the evolutionary component of biodiversity in order to maximize ecosystem function and stability (Cadotte, 2013; Cadotte et al., 2012) and ensure a sustainable provision of goods and services (Faith et al., 2010; Forest et al., 2007). The CToL could also be severely pruned if the drivers of extinction target specifically some clades, a scenario of strong phylogenetic signal in which deeper branches would be lost from the CToL (Heard & Mooers, 2000). Nonetheless, the pattern of extinction risk along a phylogeny remains debated especially for mammals (see Purvis, Agapow, Gittleman, & Mace, 2000 vs. Verde Arregoitia, Blomberg, & Fisher, 2013). For terrestrial angiosperms, however, evolutionarily young species in species‐rich (Schwartz & Simberloff, 2001) and more rapidly diversifying clades (Davies et al., 2011) are more threatened (but see Vamosi & Wilson, 2008). For cycads, all threatened species are not significantly clustered on the CToL. Also, threatened cycad species are not evolutionarily older than nonthreatened species, but there is a trend toward highly threatened species exhibiting high ED, that is, highly threatened cycad species tend to be evolutionarily older, a pattern that contrasts with what has been reported for animals [e.g., birds (Jetz et al., 2014); mammals (Verde Arregoitia et al., 2013)]. These contrasting findings echo, perhaps, the differences in evolutionary history between different taxonomic groups (plants vs. animals, angiosperm vs. gymnosperm), and the difference in tree topology (coalescent‐like topology for cycads vs. Yule and birth–death models for most taxonomic groups; Davies, 2015; Davies & Yessoufou, 2013; Mooers et al., 2012) is also one of the potential drivers. The weak phylogenetic signal in threat indicates that threatened cycads are not particularly clustered in some clades, suggesting that a clade‐based priority setting is inappropriate for cycad conservation.

However, it remains possible that prioritization based on species using ED scores might contribute significantly to safeguard most evolutionary diversity on the tree of life as previously shown (Jetz et al., 2014; Redding et al., 2008). We tested the relevance of species‐based prioritization for cycads in different ways. Unlike the loss of all VU or EN species that is not different from random loss, the loss of the top 50% ED species or all the 215 threatened cycad species or all critically endangered species would result in a greater loss of PD than expected, perhaps supporting an earlier finding that “the loss of evolutionary history with loss of species can be roughly linear” (Mooers et al., 2012). Even though we acknowledge that the loss of all threatened species may not have power to test for statistical differences, our results for ED and across threat categories suggest that ED can be used to inform the prioritization efforts of cycads for conservation and also confirm the urgent need to prioritize CR species as their loss would also prune more PD than expected.

How to prevent the loss of threatened cycads in the face of the extinction crisis and limited funds? An integrative approach that combines multiple facets of diversity analyzed within a biogeographical framework has been proposed as the best alternative to inform conservation prioritization, as no single diversity metric can be used as a silver bullet for conservation (Daru et al., 2015; Jetz, Rahbek, & Colwell, 2004; Mazel, 2014). Our findings indicate that even high‐altitude habitats (e.g., mountains) regarded as refuges for ancient lineages (Fjeldså & Lovett, 1997; Jetz et al., 2004) would not protect evolutionarily old cycads (i.e., high‐ED cycads). Also, protected areas are increasingly shown not to be efficient for conservation (Daru & le Roux, 2016; Mouillot et al., 2016). Vertebrates in particular (mammals, birds, reptiles, and amphibians) have been central to most phylogenetically informed conservation studies (Isaac et al., 2007; Jetz et al., 2014; Tonini, Beard, Ferreira, Jetz, & Pyron, 2016). Our finding that cycads are more evolutionarily distinct than vertebrates supports efforts to conserve species in this clade. Interestingly, we found that all five hotspots of cycad diversity defined in this study are within the global network of protected areas; this is also a finding unique to cycads with regard to other taxonomic groups (Daru & le Roux, 2016; Mouillot et al., 2016).

While southern Africa and eastern Australia are hotspots of species richness and PD, hotspots of species endemism are in southern Africa, northeast Australia, and some parts of the Indo‐Pacific, and hotspot of phylogenetic endemism is in Mexico. This regionalization of hotspots, which perhaps mirrors the regionalization of diversification events (as reported above), will facilitate conservation efforts as we know where geographically hotspots are located. Although all diversity hotspots are within protected areas, this can, however, be misleading simply because several high‐EDGE species are not found in protected areas [e.g., *Zamia skinneri, Zamia montana, Ceratozamia morrettii* (I.U.C.N., 2010)], and this calls for global campaign to raise public awareness of this issue, train conservation officers on EDGE concept, and design specific conservation projects for high‐EDGE species. The genera *Zamia*,* Encephalartos*,* Ceratozamia*, and *Cycas* are the most dominant numerically in the EDGE ranking and are distributed in the New World and Africa, making these two geographical regions global “hot spots” of species needing urgent attention for conservation (Isaac et al., 2007).

## 5 Conclusion

The emerging pattern in extinction risk studies indicates that threatened species are clustered on a phylogeny (Purvis et al., 2000; Yessoufou & Davies, 2016), and their loss would prune severely the branches of the tree of life (Davies, 2015). This pattern has been shown for both angiosperms and vertebrates. For gymnosperm, here cycads, we show that extinction risk is not clustered on the cycad tree of life and the loss of top ED or critically endangered species would actually prune more PD than random expectation. To safeguard multiple facets of cycad diversity hotspots, we demonstrated that a biogeographical approach is required, as different geographical regions are hotspots of different diversity facets. We also provide a species‐level prioritization option for conservation based on EDGE score. Several cycads of high score are not in any protected areas, thus calling for more efforts to prevent the cycad tree of life from being disproportionately pruned.

## Conflict of Interest

None declared.
