# Supplementary figures and images for: Integrating biogeography, threat and evolutionary data to explore extinction crisis in the taxonomic group of cycads
Source: Ecol Evol. 2017 Mar 21;7(8):2735–46. doi: 10.1002/ece3.2660 (PMC5395460; doi:10.1002/ece3.2660)

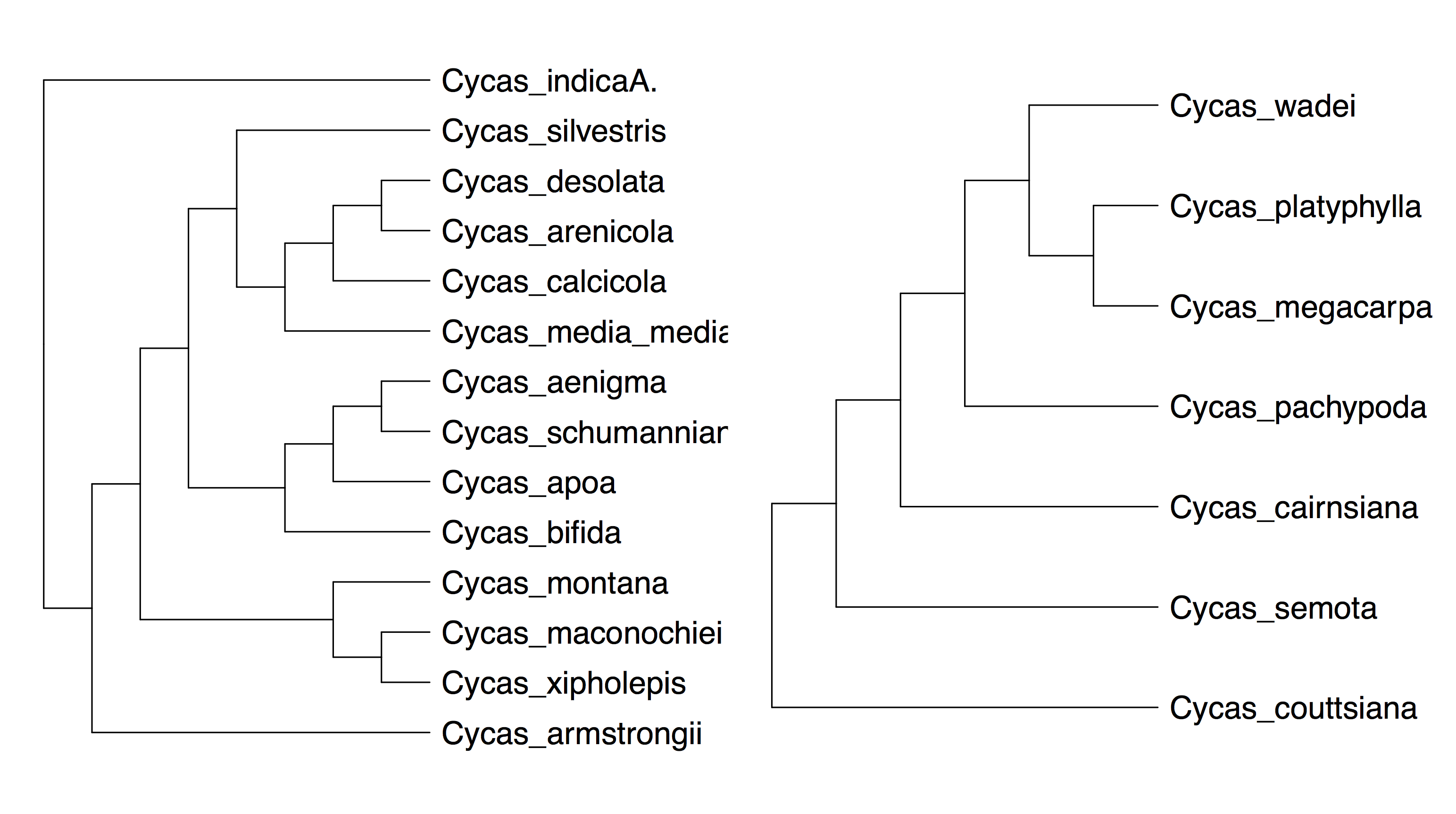

Supplement: Supplementary file 1 [file ECE3-7-2735-s001.tiff]

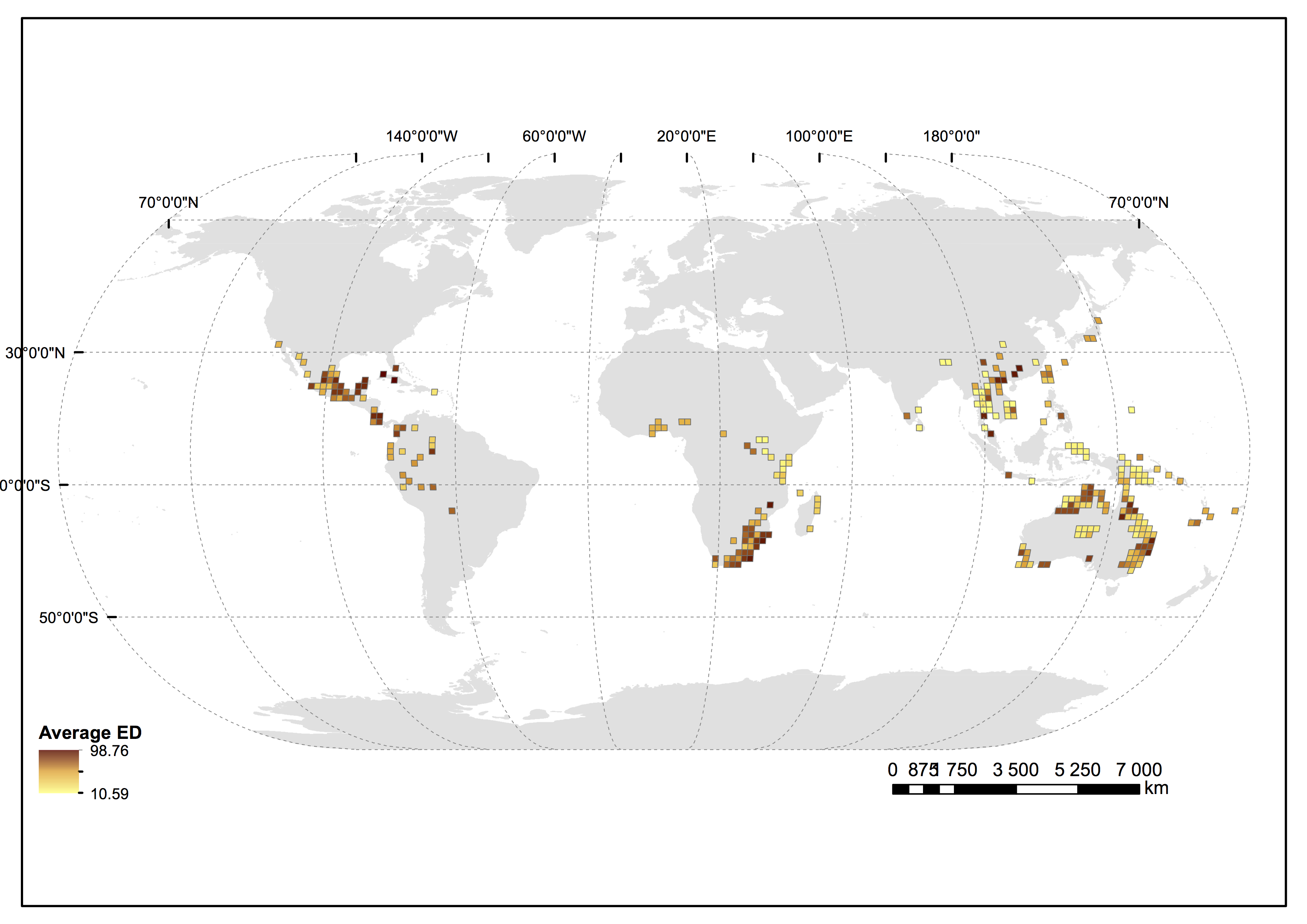

Supplement: Supplementary file 2 [file ECE3-7-2735-s002.tiff]

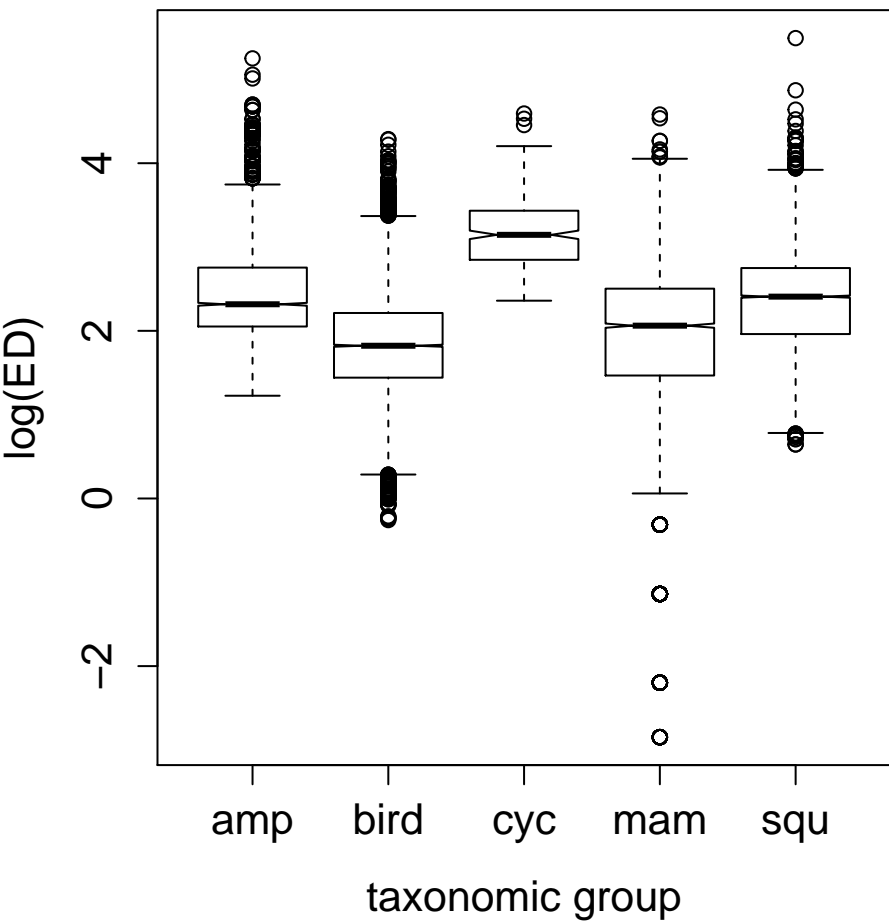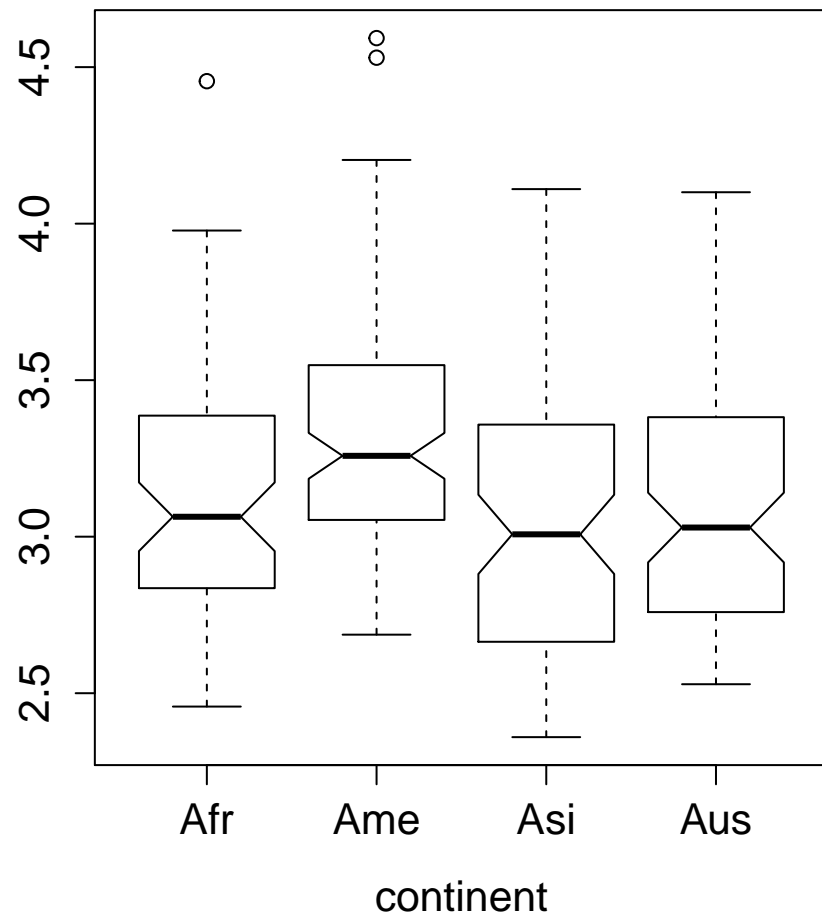

Supplement: Supplementary file 3 [file ECE3-7-2735-s003.pdf]
