# Supplementary material for: Integrating biogeography, threat and evolutionary data to explore extinction crisis in the taxonomic group of cycads
Source: Ecol Evol. 2017 Mar 21;7(8):2735–46. doi: 10.1002/ece3.2660 (PMC5395460; doi:10.1002/ece3.2660)
Supplement: Supplementary file 4 [file ECE3-7-2735-s004.doc]

*Ecology and Evolution*

**SUPPORTING INFORMATION**

**Integrating biogeography, phylogeny and threat data to explore extinction crisis in the taxonomic group of cycads**

Kowiyou Yessoufou^1^, Barnabas H. Daru^2^, Respina Tafirei^3^, Hosam O. Elansary^4,5^, and Isaac Rampedi^3^

*^1^Department of Environmental Sciences, University of South Africa, Florida campus, Florida 1710, South Africa*

*^2^Department of Plant Sciences, University of Pretoria, Pretoria, South Africa*

*^3^Department of Geography and Built Environment, University of Johannesburg, APK campus, Johannesburg, South Africa*

*^4^Department of Floriculture, Ornamental Horticulture and Garden Design, Alexandria University, Alexandria, Egypt*

*^5^Biodiversity Institute of Ontario, University of Guelph, ON N1G 2W1, Canada*

**Table S1** EDGE and ED scores of all cycad species with their geographical distribution parameters. Species are listed in decreasing order of EDGE values. Geographical parameters are spatial distribution range and altitude. Global endangerment was measured following IUCN threat categories: LC, least Concern; NT, Near Threatened; VU, Vulnerable; EN, Endangered and CR, Critically Endangered, DD, Data Deficient. NA, data non available.

| **Ranking** | **Species** | **Global Endangerment** | **ED scores (MY)** | **EDGE scores** | **Range**  **(km^2^)** | **Minimum altitude**  **(m above sea level)** | **Maximum altitude**  **(m above sea level)** |
| --- | --- | --- | --- | --- | --- | --- | --- |
| 1 | *Microcycas calocama* | CR | 98.76152685 | 7.375371 | NA | NA | NA |
| 2 | *Chigua bernalii* | CR | 92.77870776 | 7.313527 | NA | NA | NA |
| 3 | *Zamia vazquezii* | CR | 66.91129942 | 6.990938 | NA | NA | NA |
| 4 | *Encephalartos dyerianus* | CR | 47.18980637 | 6.647736 | 0.3 | NA | 700 |
| 5 | *Encephalartos inopinus* | CR | 46.26646889 | 6.62839 | NA | 600 | 800 |
| 6 | *Zamia macrochiera* | CR | 45.24071863 | 6.606449 | NA | NA | NA |
| 7 | *Ceratozamia zoquorum* | CR | 44.33579558 | 6.586686 | 40 | NA | NA |
| 8 | *Cycas szechuanensis* | CR | 42.39771664 | 6.542996 | NA | NA | NA |
| 9 | *Zamia spartea* | CR | 41.82244706 | 6.529651 | 1235 | NA | NA |
| 10 | *Ceratozamia chimalapensis* | CR | 39.54590223 | 6.475023 | NA | NA | NA |
| 11 | *Ceratozamia fuscoviridis* | CR | 39.03436558 | 6.462327 | NA | NA | NA |
| 12 | *Ceratozamia huastercorum* | CR | 39.03436558 | 6.462327 | NA | NA | NA |
| 13 | *Zamia urep* | CR | 38.73881806 | 6.454917 | 30 | NA | NA |
| 14 | *Cycas fugax* | CR | 37.99666306 | 6.436065 | NA | NA | 200 |
| 15 | *Encephalartos cupidus* | CR | 34.96229599 | 6.35506 | 58 | 700 | 1 500 |
| 16 | *Encephalartos middleburgensis* | CR | 34.96229599 | 6.35506 | NA | 1 100 | 1 400 |
| 17 | *Zamia hymenophyllidia* | CR | 34.88072687 | 6.352789 | NA | NA | NA |
| 18 | *Zamia imperialis* | CR | 34.74486641 | 6.348995 | NA | NA | NA |
| 19 | *Encephalartos whitelockii* | CR | 34.4420844 | 6.340489 | NA | 1 000 | 1 300 |
| 20 | *Cycas curranii* | CR | 34.03290306 | 6.328876 | NA | NA | NA |
| 21 | *Ceratozamia decumbens* | CR | 33.73551899 | 6.320351 | NA | NA | NA |
| 22 | *Ceratozamia santillanii* | CR | 33.73551899 | 6.320351 | NA | NA | NA |
| 23 | *Zamia prasina* | CR | 33.45520758 | 6.312249 | NA | NA | NA |
| 24 | *Zamia monticola* | CR | 31.85847478 | 6.264798 | NA | NA | NA |
| 25 | *Zamia onan-reyesii* | CR | 31.72164993 | 6.260626 | NA | NA | NA |
| 26 | *Zamia tolimensis* | CR | 31.59700529 | 6.256809 | NA | NA | NA |
| 27 | *Zamia nesophila* | CR | 31.01836428 | 6.238898 | NA | NA | NA |
| 28 | *Encephalartos dolomiticus* | CR | 30.92972888 | 6.236126 | NA | 1 100 | 1 500 |
| 29 | *Ceratozamia zaragozae* | CR | 30.23118189 | 6.214006 | 45 | NA | NA |
| 30 | *Cycas taiwaniana* | EN | 60.36197811 | 6.196232 | NA | 400 | 1 100 |
| 31 | *Dioon caputoi* | EN | 60.02894657 | 6.19079 | NA | NA | NA |
| 32 | *Zamia hamannii* | CR | 29.18056715 | 6.179787 | NA | NA | NA |
| 33 | *Dioon spinulosum* | EN | 58.1311588 | 6.170667 | NA | NA | NA |
| 34 | *Zamia decumbens* | CR | 28.15189141 | 6.145109 | NA | NA | NA |
| 35 | *Cycas hongheensis* | CR | 28.09396076 | 6.143119 | NA | 400 | 600 |
| 36 | *Zamia wallisii* | CR | 28.07088709 | 6.142326 | NA | NA | NA |
| 37 | *Macrozamia cranei* | EN | 54.6574816 | 6.098658 | NA | 400 | 600 |
| 38 | *Encephalartos aemulans* | CR | 26.60506999 | 6.090588 | NA | 600 | 1 100 |
| 39 | *Zamia inermis* | CR | 26.59267689 | 6.090139 | NA | NA | NA |
| 40 | *Zamia gentry* | CR | 26.08392961 | 6.071529 | 5 | NA | NA |
| 41 | *Cycas kuesteriana* | CR | 25.53290348 | 6.050974 | NA | NA | NA |
| 42 | *Zamia pyrophylla* | CR | 25.36689544 | 6.044698 | NA | NA | NA |
| 43 | *Encephalartos pterogononus* | CR | 24.90164089 | 6.026895 | 35 | 700 | 1 000 |
| 44 | *Encephalartos laevifolius* | CR | 24.40140887 | 6.007393 | NA | 950 | 1 800 |
| 45 | *Zamia pygmaea* | CR | 24.13964507 | 5.997035 | NA | NA | NA |
| 46 | *Zamia montana* | CR | 23.94413954 | 5.989228 | NA | NA | NA |
| 47 | *Zamia purpuria* | CR | 23.94413954 | 5.989228 | NA | NA | NA |
| 48 | *Zamia skinneri* | EN | 47.67761744 | 5.964661 | 6250 | NA | NA |
| 49 | *Encephalartos hirsutus* | CR | 23.22916216 | 5.960146 | NA | 800 | 1 000 |
| 50 | *Ceratozamia morettii* | EN | 47.26377399 | 5.956123 | 10 | NA | NA |
| 51 | *Ceratozamia miqueliana* | CR | 22.91371599 | 5.947041 | NA | NA | NA |
| 52 | *Encephalartos cerinus* | CR | 22.75749149 | 5.940487 | NA | 500 | 900 |
| 53 | *Encephalartos latifrons* | CR | 22.23855256 | 5.918401 | NA | 200 | 600 |
| 54 | *Zamia amplifola* | CR | 21.18935565 | 5.872201 | NA | NA | NA |
| 55 | *Ceratozamia mexeorum* | EN | 42.82402982 | 5.859624 | 25 | NA | NA |
| 56 | *Stangeria eriopus* | VU | 86.06691786 | 5.852971 | NA | 10 | 750 |
| 57 | *Dioon stevensonii* | CR | 20.61607388 | 5.846026 | NA | NA | NA |
| 58 | *Cycas annaikalensis* | CR | 20.23621295 | 5.828297 | NA | NA | 940 |
| 59 | *Cycas zambalensis* | CR | 20.19838475 | 5.826514 | NA | NA | NA |
| 60 | *Ceratozamia euryphyllidia* | CR | 20.08057361 | 5.820941 | NA | NA | NA |
| 61 | *Ceratozamia hondurensis* | CR | 19.85266019 | 5.81007 | NA | NA | NA |
| 62 | *Ceratozamia alvarezii* | EN | 39.54590223 | 5.781876 | 16 | NA | NA |
| 63 | *Cycas pachypoda* | CR | 19.21076297 | 5.778804 | NA | NA | NA |
| 64 | *Cycas wadei* | CR | 19.06554934 | 5.771593 | NA | 20 | 50 |
| 65 | *Zamia lacandona* | EN | 38.76276788 | 5.762373 | 3 400 | NA | NA |
| 66 | *Zamia variegate* | EN | 36.92315299 | 5.715003 | NA | NA | NA |
| 67 | *Encephalartos heenanii* | CR | 17.77268043 | 5.704991 | 300 | 750 | 1 750 |
| 68 | *Encephalartos msinganus* | CR | 17.77268043 | 5.704991 | 10 | 900 | 1 200 |
| 69 | *Ceratozamia hildae* | EN | 36.39391589 | 5.70095 | NA | NA | NA |
| 70 | *Cycas cantafolia* | CR | 17.23081084 | 5.675702 | NA | NA | NA |
| 71 | *Zamia katzeriana* | EN | 34.87334824 | 5.659436 | NA | NA | NA |
| 72 | *Ceratozamia mirandae* | EN | 34.77023182 | 5.656558 | NA | NA | NA |
| 73 | *Cycas hainanensis* | EN | 34.03290306 | 5.635729 | NA | 0 | 1200 |
| 74 | *Zamia furfuracea_A* | EN | 33.70254139 | 5.626254 | 630 | NA | NA |
| 75 | *Zamia disodon* | CR | 16.34563319 | 5.62593 | NA | NA | NA |
| 76 | *Zamia restrepoi* | CR | 16.34563319 | 5.62593 | NA | 75 | 150 |
| 77 | *Macrozamia elegans* | EN | 33.38726199 | 5.617128 | 112 | 120 | 150 |
| 78 | *Cycas tansachana* | CR | 15.77084129 | 5.59223 | 10 | NA | 400 |
| 79 | *Cycas chamoensis* | CR | 14.78945112 | 5.531931 | NA | NA | NA |
| 80 | *Zamia kickxii* | CR | 14.68817057 | 5.525496 | NA | NA | NA |
| 81 | *Ceratozamia becerrae* | EN | 30.23118189 | 5.520859 | 1 000 | NA | NA |
| 82 | *Cycas elephantipes* | EN | 29.13189834 | 5.485026 | NA | NA | NA |
| 83 | *Cycas changjiangensis* | EN | 29.0368105 | 5.481865 | NA | 600 | 800 |
| 84 | *Cycas javana* | EN | 28.43630201 | 5.46167 | NA | NA | NA |
| 85 | *Encephalartos eugenemaraisii* | EN | 28.26662054 | 5.455889 | NA | 1 400 | 1 500 |
| 86 | *Encephalartos umbeluziensis* | EN | 28.26662054 | 5.455889 | 336 | 50 | 120 |
| 87 | *Zamia dressleri* | EN | 28.07088709 | 5.449179 | 2 530 | NA | NA |
| 88 | *Encephalartos munchii* | CR | 13.48623715 | 5.445788 | 3 | 1 000 | 1 100 |
| 89 | *Zamia lucayana* | EN | 27.68496665 | 5.435815 | 13 | NA | NA |
| 90 | *Zamia meermanii* | EN | 27.52500143 | 5.430222 | NA | NA | NA |
| 91 | *Encephalartos equatorialis* | CR | 12.85206894 | 5.401023 | 5 | NA | 1 000 |
| 92 | *Zamia ipentiensis* | EN | 26.52220215 | 5.394435 | 50 | NA | NA |
| 93 | *Encephalartos ngoyanus* | VU | 53.41135673 | 5.382867 | NA | 200 | 600 |
| 94 | *Zamia picta* | EN | 25.88300299 | 5.370936 | NA | NA | NA |
| 95 | *Zamia fischeri* | EN | 25.79943714 | 5.367822 | 2770 | NA | NA |
| 96 | *Zamia herrerae* | VU | 52.42318283 | 5.364539 | NA | NA | NA |
| 97 | *Encephalartos gratus* | VU | 52.39442418 | 5.364001 | NA | 650 | 900 |
| 98 | *Cycas taitungensis* | EN | 25.68127028 | 5.363403 | 65 | 400 | 900 |
| 99 | *Macrozamia spiralis* | EN | 25.61487566 | 5.360912 | NA | NA | NA |
| 100 | *Cycas beddomei* | EN | 25.56325424 | 5.35897 | 388 | 300 | 900 |
| 101 | *Cycas circinalis* | EN | 25.56325424 | 5.35897 | NA | 300 | 1000 |
| 102 | *Ceratozamia sabatoi* | EN | 25.53290346 | 5.357827 | NA | NA | NA |
| 103 | *Ceratozamia matudae* | EN | 24.96245108 | 5.336093 | 5 000 | NA | NA |
| 104 | *Cycas candida* | EN | 24.93180331 | 5.334912 | 55 | NA | NA |
| 105 | *Dioon califanoi* | EN | 2465399859 | 5.324141 | 126 | NA | NA |
| 106 | *Cycas hoabinhensis* | EN | 24.43045531 | 5.315389 | NA | 50 | 150 |
| 107 | *Encephalartos sclavoi* | CR | 11.67276377 | 5.315389 | NA | 1 800 | 2 100 |
| 108 | *Zamia neurophyllidia* | VU | 48.38625065 | 5.285966 | NA | NA | NA |
| 109 | *Macrozamia flexuosa* | EN | 22.74607533 | 5.246859 | NA | NA | NA |
| 110 | *Cycas debaoensis* | CR | 10.80661968 | 5.241249 | NA | 300 | 1300 |
| 111 | *Macrozamia humilis* | VU | 45.99115911 | 5.236254 | NA | NA | 600 |
| 112 | *Dioon sonorense* | EN | 22.43077809 | 5.233492 | NA | NA | NA |
| 113 | *Cycas macrocarpa* | VU | 45.82548691 | 5.232722 | NA | NA | NA |
| 114 | *Ceratozamia norstogii* | EN | 22.39374741 | 5.23191 | 1 100 | NA | NA |
| 115 | *Zamia furfuracea_B* | EN | 21.53129458 | 5.194347 | 631 | NA | NA |
| 116 | *Cycas elongate* | EN | 21.47247138 | 5.191733 | NA | 50 | 200 |
| 117 | *Dioon holmgrenii* | EN | 21.43384545 | 5.190012 | NA | NA | NA |
| 118 | *Cycas pranburiensis* | VU | 43.33354594 | 5.178036 | NA | 5 | 30 |
| 119 | *Dioon rzedowskii* | EN | 20.82433129 | 5.162467 | 25 | NA | NA |
| 120 | *Ceratozamia latifolia* | EN | 20.70880668 | 5.15716 | NA | NA | NA |
| 121 | *Ceratozamia robusta* | EN | 20.70880668 | 5.15716 | NA | NA | NA |
| 122 | *Ceratozamia microstrobila* | VU | 41.76928923 | 5.142115 | 1 000 | NA | NA |
| 123 | *Cycas lindstromii* | EN | 20.23621295 | 5.135149 | 4280 | 0 | 30 |
| 124 | *Encephalartos lebomboensis* | EN | 20.10179349 | 5.1288 | NA | 500 | 1 000 |
| 125 | *Ceratozamia whitelockiana* | EN | 19.85266019 | 5.116923 | NA | NA | NA |
| 126 | *Zamia elegantissima* | EN | 19.60962709 | 5.1052 | 100 | NA | NA |
| 127 | *Zamia cunaria* | VU | 39.96309189 | 5.098966 | 3140 | NA | NA |
| 128 | *Macrozamia pauliguilielmi* | EN | 18.55765384 | 5.052825 | NA | 5 | 25 |
| 129 | *Cycas platyphylla* | EN | 18.53765384 | 5.051785 | NA | 400 | 750 |
| 130 | *Cycas inermis* | VU | 37.99666306 | 5.04977 | NA | NA | NA |
| 131 | *Cycas multipinnata* | EN | 18.43632098 | 5.046585 | 27 040 | 200 | 1300 |
| 132 | *Cycas micholitzii* | VU | 36.43867597 | 5.008999 | NA | 130 | 600 |
| 133 | *Cycas segmentifida* | VU | 36.43867597 | 5.008999 | NA | 600 | 900 |
| 134 | *Encephalartos concinnus* | EN | 17.17823109 | 4.979666 | NA | 800 | 900 |
| 135 | *Zamia portoricensis* | EN | 16.55332369 | 4.944685 | 220 | NA | NA |
| 136 | *Zamia creminophila* | EN | 16.38093259 | 4.934815 | 53 | NA | NA |
| 137 | *Zamia melanorrhachis* | EN | 16.38093259 | 4.934815 | NA | NA | NA |
| 138 | *Macrozamia machinii* | VU | 33.38726199 | 4.923981 | 460 | 320 | 460 |
| 139 | *Macrozamia plurinervia* | EN | 15.77149983 | 4.899123 | NA | NA | NA |
| 140 | *Zamia acuminate* | VU | 32.43503846 | 4.895899 | NA | NA | NA |
| 141 | *Macrozamia crassifolia* | VU | 32.28496411 | 4.8914 | 160 | 340 | 420 |
| 142 | *Macrozamia conferta* | VU | 32.28496411 | 4.8914 | 423 | 600 | 750 |
| 143 | *Macrozamia viridis* | EN | 15.17074349 | 4.862645 | 1 000 | NA | NA |
| 144 | *Zamia standleyi* | VU | 31.01836428 | 4.852604 | NA | NA | NA |
| 145 | *Cycas panzhihuaensis* | VU | 30.05926101 | 4.822191 | 14 500 | 1 100 | 2000 |
| 146 | *Cycas balansae* | NT | 60.96541261 | 4.819724 | NA | 100 | 800 |
| 147 | *Cycas collina* | VU | 29.8268445 | 4.81468 | 20 000 | 400 | 900 |
| 148 | *Cycas lacrimans* | EN | 14.35800448 | 4.811078 | NA | NA | NA |
| 149 | *Cycas conferta* | NT | 60.36197811 | 4.809938 | NA | NA | NA |
| 150 | *Cycas riuminiana* | EN | 14.32453779 | 4.808897 | NA | 615 | 800 |
| 151 | *Encephalartos chimanimaniensis* | EN | 14.30221365 | 4.807439 | NA | NA | 1 000 |
| 152 | *Cycas armstrongii* | VU | 29.42156612 | 4.801446 | NA | NA | NA |
| 153 | *Encephalartos horridus* | EN | 13.80458849 | 4.781111 | NA | 100 | 400 |
| 154 | *Encephalartos macrostrobilus* | EN | 13.78089777 | 4.772777 | 50 | 900 | 1 400 |
| 155 | *Encephalartos angustifolia* | VU | 28.15189141 | 4.758814 | 9 000 | NA | NA |
| 156 | *Encephalartos delucanus* | EN | 13.48623715 | 4.752641 | NA | 1 200 | 1 950 |
| 157 | *Encephalartos marunguensis* | VU | 27.0007844 | 4.718527 | 7 500 | 1 400 | 1 700 |
| 158 | *Encephalartos arenarius* | EN | 12.5367299 | 4.707876 | 450 | 100 | 200 |
| 159 | *Encephalartos caffer* | NT | 53.41135673 | 4.68972 | NA | 300 | 700 |
| 160 | *Macrozamia lomandroides* | EN | 12.5367299 | 4.684848 | NA | NA | NA |
| 161 | *Cycas condaoensis* | VU | 26.07318304 | 4.684838 | 20 | NA | NA |
| 162 | *Cycas chamberlainii* | EN | 12.51480679 | 4.683227 | NA | 615 | 800 |
| 163 | *Ceratozamia vovidesii* | VU | 25.25.92662591 | 4.67941 | NA | 1 000 | 1 700 |
| 164 | *Zamia encephalartoides* | VU | 25.66209454 | 4.669537 | 266 | NA | NA |
| 165 | *Zamia gomeziana* | VU | 25.36689544 | 4.658404 | NA | NA | NA |
| 166 | *Cycas brachycantha* | NT | 51.24649223 | 4.64912 | NA | NA | NA |
| 167 | *Cycas tropophylla* | NT | 51.24649223 | 4.64912 | 400 | NA | NA |
| 168 | *Macrozamia secunda* | VU | 24.76861886 | 4.635452 | NA | NA | NA |
| 169 | *Encephalartos humilis* | VU | 24.40140887 | 4.621099 | NA | NA | NA |
| 170 | *Encephalartos kisambo* | EN | 11.67276377 | 4.618897 | NA | 800 | 1 800 |
| 171 | *Encephalartos paucidentatis* | VU | 23.34009366 | 4.578419 | 424 | 1 000 | 1 500 |
| 172 | *Cycas terryana* | VU | 23.34009366 | 4.576505 | NA | NA | NA |
| 173 | *Encephalartos princeps* | VU | 22.75749149 | 4.554192 | 1 870 | 200 | 800 |
| 174 | *Dioon merolae* | VU | 22.43077809 | 4.540345 | NA | NA | NA |
| 175 | *Cycas micronesica* | EN | 10.58724336 | 4.529346 | 11250000 | NA | NA |
| 176 | *Cycas seemani* | VU | 21.69838755 | 4.508588 | NA | 0 | 600 |
| 177 | *Zamia soconuscensis* | VU | 21.53129458 | 4.5012 | NA | NA | NA |
| 178 | *Dioon purpusii* | VU | 21.43384545 | 4.496865 | NA | 1 000 | 1 500 |
| 179 | *Encephalartos altensteinii* | VU | 21.40559109 | 4.495605 | NA | 0 | 600 |
| 180 | *Cycas cairnsiana* | VU | 21.22964922 | 4.487721 | NA | 450 | 500 |
| 181 | *Cycas ferruginea* | NT | 43.33354594 | 4.484889 | 7215 | NA | NA |
| 182 | *Encephalartos barteri* | VU | 21.02188177 | 4.478331 | NA | 400 | 1 400 |
| 183 | *Dioon argenteum* | VU | 20.82433129 | 4.46932 | 350 | 1 100 | 1 600 |
| 184 | *Cycas silvestris* | VU | 20.74771168 | 4.465803 | NA | NA | NA |
| 185 | *Cycas cupida* | VU | 20.616169 | 4.459736 | 60 | NA | NA |
| 186 | *Encephalartos senticosus* | VU | 20.10179349 | 4.435652 | NA | 300 | 800 |
| 187 | *Ceratozamia Mexicana* | VU | 20.08057261 | 4.434646 | NA | NA | NA |
| 188 | *Encephalartos schmitzii* | VU | 19.82233343 | 4.42232 | NA | 1 000 | 1 400 |
| 189 | *Encephalartos ghellincki* | VU | 19.81469597 | 4.421954 | NA | 700 | 2 400 |
| 190 | *Cycas nongnoochiae* | VU | 19.79711088 | 4.421108 | NA | 50 | 100 |
| 191 | *Cycas tuckeri* | VU | 19.79711088 | 4.421108 | 15 | NA | NA |
| 192 | *Cycas saxatilis* | VU | 19.20190975 | 4.392072 | NA | NA | NA |
| 193 | *Encephalartos aplanatus* | VU | 19.12329106 | 4.388172 | 295 | 100 | 600 |
| 194 | *Dioon angustifolium* | VU | 18.87281058 | 4.375647 | NA | NA | NA |
| 195 | *Dioon tomasellii* | VU | 18.87281058 | 4.375647 | NA | 600 | 1 850 |
| 196 | *Zamia loddigesii* | NT | 38.73881806 | 4.375476 | NA | NA | NA |
| 197 | *Cycas bifida* | VU | 18.72093951 | 4.367975 | NA | 100 | 300 |
| 198 | *Cycas megacarpa* | VU | 18.53765384 | 4.358638 | NA | 150 | 300 |
| 199 | *Cycas aculeate* | VU | 18.22700347 | 4.34261 | 10 | NA | NA |
| 200 | *Cycas pectinata_B* | VU | 17.56072929 | 4.307342 | NA | 600 | 1 300 |
| 201 | *Zamia oreillyi* | VU | 17.15521714 | 4.285252 | NA | NA | NA |
| 202 | *Encephalartos ferox* | NT | 35.18108407 | 4.281684 | NA | 20 | 100 |
| 203 | *Zamia pseudoparasitica* | NT | 34.88072687 | 4.273347 | NA | NA | NA |
| 204 | *Encephalartos manikensis* | VU | 16.90228432 | 4.271223 | NA | 600 | 1 400 |
| 205 | *Encephalartos lanatus* | NT | 34.20526447 | 4.254343 | NA | 1 200 | 1 500 |
| 206 | *Zamia stricta* | VU | 16.55332369 | 4.251538 | 25 | NA | NA |
| 207 | *Cycas desolata* | VU | 16.41264168 | 4.243491 | NA | 450 | 550 |
| 208 | *Zamia lawsoniana* | NT | 33.70254139 | 4.23996 | NA | NA | NA |
| 209 | *Macrozamia platyrhachis* | VU | 15.78571553 | 4.206823 | NA | NA | NA |
| 210 | *Zamia integrifolia* | NT | 32.54882493 | 4.206149 | NA | NA | NA |
| 211 | *Macrozamia fawcettii* | NT | 32.37059711 | 4.200822 | 5 500 | 5 | 550 |
| 212 | *Cycas couttsiana* | NT | 31.82361505 | 4.184295 | NA | NA | 700 |
| 213 | *Macrozamia occidua* | VU | 15.18300583 | 4.170256 | 10 | 800 | 1 000 |
| 214 | *Macrozamia cardiacensis* | VU | 15.18194666 | 4.170191 | 14 | 500 | 640 |
| 215 | *Macrozamia parcifolia* | VU | 15.17074349 | 4.169498 | NA | 60 | 220 |
| 216 | *Encephalartos longifolius* | NT | 30.93709813 | 4.156915 | NA | 200 | 700 |
| 217 | *Cycas ophiolitica* | VU | 14.90585325 | 4.152982 | NA | 150 | 250 |
| 218 | *Cycas semota* | NT | 30.74310522 | 4.150823 | NA | NA | NA |
| 219 | *Zamia incognita* | VU | 14.74109482 | 4.142569 | NA | NA | NA |
| 220 | *Zamia amblyphyllidia* | VU | 14.68817052 | 4.139201 | NA | NA | NA |
| 221 | *Cycas brunnea* | NT | 30.05926101 | 4.129044 | NA | NA | NA |
| 222 | *Cycas simplicipinna* | NT | 29.8268445 | 4.121533 | NA | 600 | 1 300 |
| 223 | *Cycas nathorstii* | VU | 14.35800448 | 4.117931 | NA | 30 | 300 |
| 224 | *Cycas sexseminifera* | VU | 14.11838029 | 4.102206 | NA | NA | NA |
| 225 | *Zamia manicata* | NT | 29.18056715 | 4.100345 | NA | NA | NA |
| 226 | *Encephalartos trispinosus* | VU | 13.90458849 | 4.087963 | NA | 100 | 600 |
| 227 | *Cycas vespertilio* | NT | 28.43630201 | 4.075376 | NA | NA | NA |
| 228 | *Cycas pectinata_A* | VU | 13.66021178 | 4.071432 | NA | 600 | 1 300 |
| 229 | *Zamia tuerckeimii* | NT | 27.68496665 | 4.04952 | NA | NA | NA |
| 230 | *Zamia chigua* | NT | 27.52500143 | 4.043928 | NA | NA | NA |
| 231 | *Encephalartos ituriensis* | NT | 27.0007844 | 4.02538 | NA | 1 100 | 1 200 |
| 232 | *Macrozamia johnsonii* | LC | 54.6574816 | 4.019217 | 222 | NA | NA |
| 233 | *Zamia boliviana* | NT | 26.80183661 | 4.018249 | NA | NA | NA |
| 234 | *Encephalartos schaijesii* | VU | 12.5674151 | 3.993965 | NA | 1 450 | 1 500 |
| 235 | *Zamia paucijuga* | NT | 26.08392961 | 3.992088 | NA | NA | NA |
| 236 | *Cycas shanyaensis* | VU | 12.51480679 | 3.99008 | 10 | 700 | 800 |
| 237 | *Zamia sandovalii* | NT | 25.79943714 | 3.981528 | NA | NA | NA |
| 238 | *Zamia pumila* | NT | 25.78334669 | 3.980927 | NA | NA | NA |
| 239 | *Cycas nitida* | NT | 25.68127028 | 3.977109 | NA | NA | NA |
| 240 | *Encephalartos mackenziei* | NT | 24. 78281687 | 3.942855 | NA | 1 800 | 2 000 |
| 241 | *Cycas bougainvilleana* | NT | 24.69741468 | 3.939538 | NA | NA | NA |
| 242 | *Bowenia serrulata* | LC | 50.14995324 | 3.934762 | NA | 30 | 150 |
| 243 | *Bowenia spectabilis* | LC | 50.14995324 | 3.934762 | NA | 0 | 750 |
| 244 | *Zamia fairchildiana* | NT | 24.51522014 | 3.932422 | NA | NA | NA |
| 245 | *Cycas petraea* | NT | 24.43045531 | 3.929095 | 60 | NA | NA |
| 246 | *Zamia ulei* | NT | 24.13964507 | 3.917593 | NA | NA | NA |
| 247 | *Cycas siamensis* | VU | 11.14556768 | 3.883259 | NA | NA | 300 |
| 248 | *Macrozamia longispina* | NT | 23.2755697 | 3.882618 | 50 | 200 | 700 |
| 249 | *Encephalartos cycadifolius* | LC | 47.18980637 | 3.875148 | 290 | 1 200 | 1 800 |
| 250 | *Cycas diannanensis* | VU | 11.02611836 | 3.873375 | NA | 600 | 1 800 |
| 251 | *Cycas falcate* | VU | 11.02611836 | 3.873375 | 1350 | NA | NA |
| 252 | *Zamia lecointei* | NT | 22.97656965 | 3.870224 | NA | NA | NA |
| 253 | *Macrozamia stenomera* | NT | 22.74607533 | 3.860564 | NA | NA | NA |
| 254 | *Cycas guizhouensis* | VU | 10.80661968 | 3.854955 | NA | 400 | 1300 |
| 255 | *Cycas montana* | NT | 22.60174471 | 3.854468 | NA | NA | NA |
| 256 | *Cycas zeylanica* | VU | 10.58724336 | 3.836199 | NA | 5 | 50 |
| 257 | *Encephalartos turneri* | LC | 45.29298688 | 3.83499 | NA | 600 | 1 200 |
| 258 | *Zamia pseudomonticola* | NT | 21.74730214 | 3.817594 | NA | NA | NA |
| 259 | *Cycas chevalieri* | NT | 21.6838055 | 3.814798 | NA | NA | NA |
| 260 | *Zamia lindenii* | NT | 21.50498881 | 3.806884 | NA | NA | NA |
| 261 | *Cycas papua* | NT | 21.47898495 | 3.805728 | NA | NA | NA |
| 262 | *Encephalartos natalensis* | NT | 21.40559109 | 3.802458 | NA | 200 | 1 200 |
| 263 | *Zamia muricata* | NT | 21.18935565 | 3.79276 | NA | NA | NA |
| 264 | *Lepidozamia hopei* | LC | 43.35002121 | 3.792113 | NA | 0 | 1 000 |
| 265 | *Lepidozamia peroffskyana* | LC | 43.35002121 | 3.792113 | NA | 0 | 1 000 |
| 266 | *Encephalartos lehmannii* | NT | 21.01598319 | 3.784916 | NA | 400 | 1 000 |
| 267 | *Cycas tanqingii* | NT | 20.616169 | 3.766589 | 80 | NA | 800 |
| 268 | *Dioon edule* | NT | 20.61607388 | 3.766584 | NA | NA | NA |
| 269 | *Encephalartos fridericiguilielmi* | NT | 19.81469597 | 3.728806 | NA | 700 | 1 400 |
| 270 | *Zamia obliqua* | NT | 19.60967709 | 3.718905 | NA | NA | NA |
| 271 | *Cycas campestris* | NT | 19.20190975 | 3.698924 | 20 000 | NA | NA |
| 272 | *Zamia poeppigiana* | NT | 19.07345031 | 3.692545 | NA | NA | NA |
| 273 | *Cycas dolichphylla* | NT | 18.87450817 | 3.682585 | NA | NA | NA |
| 274 | *Zamia amazonum* | NT | 18.78899749 | 3.678273 | NA | NA | NA |
| 275 | *Cycas badensis* | NT | 17.56072929 | 3.614195 | NA | NA | NA |
| 276 | *Encephalartos laurentianus* | NT | 17.44729335 | 3.608065 | NA | 450 | 550 |
| 277 | *Zamia roezlii* | NT | 17.15521714 | 3.592105 | NA | NA | NA |
| 278 | *Encephalartos hildebrandtii* | NT | 16.6308956 | 3.5628 | NA | 0 | 600 |
| 279 | *Cycas arenicola* | NT | 16.41264168 | 3.550344 | NA | NA | NA |
| 280 | *Macrozamia moorei* | NT | 16.16683711 | 3.536127 | NA | 300 | 500 |
| 281 | *Cycas anhemica* | LC | 32.74662295 | 3.51888 | NA | NA | NA |
| 282 | *Cycas yorkiana* | NT | 15.78572935 | 3.513676 | 11 530 | NA | NA |
| 283 | *Cycas apoa* | NT | 15.77197351 | 3.512856 | NA | NA | NA |
| 284 | *Encephalartos bubalinus* | NT | 15.6287761 | 3.504282 | NA | 1 300 | 2 150 |
| 285 | *Macrozamia serpentine* | NT | 15.18194666 | 3.477043 | 850 | NA | NA |
| 286 | *Encephalartos poggei* | LC | 30.92972888 | 3.463538 | NA | 500 | 1 000 |
| 287 | *Macrozamia fraseri* | LC | 29.77537824 | 3.426715 | NA | NA | NA |
| 288 | *Cycas rumphii* | NT | 14.37566603 | 3.425933 | NA | 10 | 200 |
| 289 | *Cycas scratchleyana* | NT | 14.13015235 | 3.409837 | NA | 5 | 900 |
| 290 | *Cycas pruinosa* | LC | 29.13189834 | 3.405584 | NA | NA | NA |
| 291 | *Encephalartos septentrionalis* | NT | 13.78089777 | 3.386483 | NA | 500 | 2 500 |
| 292 | *Cycas orientis* | LC | 28.09396076 | 3.370531 | NA | NA | NA |
| 293 | *Macrozamia dyeri* | LC | 28.07961636 | 3.370037 | NA | NA | NA |
| 294 | *Cycas edentate* | NT | 13.48672619 | 3.36638 | 1 000 | NA | NA |
| 295 | *Macrozamia mountperriensis* | LC | 27.25840935 | 3.341391 | NA | 50 | 400 |
| 296 | *Cycas schumanniana* | NT | 12.87560451 | 3.323219 | NA | NA | 1 600 |
| 297 | *Macrozamia douglasii* | LC | 26.54739011 | 3.315908 | NA | 0 | 150 |
| 298 | *Macrozamia lucida* | LC | 26.54739011 | 3.315908 | NA | 30 | 600 |
| 299 | *Cycas litoralis* | NT | 12.58177486 | 3.301876 | 1000 | NA | NA |
| 300 | *Macrozamia glaucophylla* | LC | 24.76861886 | 3.249157 | NA | NA | NA |
| 301 | *Macrozamia diplomera* | LC | 24.60611978 | 3.242831 | NA | NA | 500 |
| 302 | *Cycas revolute* | LC | 23.29359995 | 3.190213 | NA | 0 | 300 |
| 303 | *Encephalartos transvenosus* | LC | 23.22916216 | 3.187557 | NA | 600 | 1 500 |
| 304 | *Dioon mejiae* | LC | 22.89612329 | 3.173716 | NA | NA | NA |
| 305 | *Cycas miquellii* | LC | 21.02825668 | 3.092326 | NA | 0 | 300 |
| 306 | *Encephalartos villosus* | LC | 19.12329106 | 3.001878 | NA | 100 | 600 |
| 307 | *Cycas thouarsii* | LC | 18.70990103 | 2.981121 | NA | 0 | 200 |
| 308 | *Macrozamia polymorpha* | LC | 18.5579877 | 2.973384 | NA | NA | NA |
| 309 | *Cycas canalis* | LC | 18.22700347 | 2.956316 | NA | NA | NA |
| 310 | *Cycas media* | LC | 17.49625418 | 2.917568 | NA | 0 | 860 |
| 311 | *Macrozamia reidlei* | LC | 17.26146916 | 2.904793 | NA | NA | NA |
| 312 | *Cycas calcicola* | LC | 17.06050018 | 2.893727 | NA | 123 | 155 |
| 313 | *Cycas maconochiei* | LC | 15.93740571 | 2.829525 | NA | 0 | 40 |
| 314 | *Cycas xipholepis* | LC | 15.93740571 | 2.829525 | NA | NA | NA |
| 315 | *Cycas media ensata* | LC | 15.78572935 | 2.820529 | NA | 0 | 860 |
| 316 | *Macrozamia communis* | LC | 15.78571553 | 2.820528 | NA | 0 | 300 |
| 317 | *Cycas angulate* | LC | 15.77084129 | 2.819642 | NA | 0 | 30 |
| 318 | *Macrozamia heteromera* | LC | 15.27230078 | 2.789464 | NA | NA | 200 |
| 319 | *Macrozamia concinna* | LC | 15.18300583 | 2.783962 | NA | 800 | 1 100 |
| 320 | *Macrozamia macdonnelli* | LC | 14.90585325 | 2.773662 | NA | NA | NA |
| 321 | *Cycas basaltca* | LC | 14.90585325 | 2.766687 | NA | 230 | 260 |
| 322 | *Cycas lane-poolei* | LC | 14.37566603 | 2.732786 | NA | 300 | 370 |
| 323 | *Cycas sundaica* | LC | 14.11838029 | 2.715911 | NA | NA | NA |
| 324 | *Macrozamia montana* | LC | 13.91429658 | 2.70232 | NA | NA | NA |
| 325 | *Macrozamia reducta* | LC | 13.91429658 | 2.70232 | NA | NA | NA |
| 326 | *Encephalartos tegulaneus* | LC | 13.12263127 | 2.647779 | NA | 1 400 | 2 300 |
| 327 | *Macrozamia fearnsidei* | LC | 13.06858849 | 2.643945 | NA | 300 | 600 |
| 328 | *Macrozamia miquelii* | LC | 13.06858849 | 2.643945 | NA | 0 | 500 |
| 329 | *Macrozamia macleayi* | LC | 12.5367299 | 2.605407 | NA | 100 | 500 |
| 330 | *Cycas clivicola* | LC | 11.14556768 | 2.496964 | 300 000 | NA | 60 |
| 331 | *Cycas_sphaerica* | DD | 17.23081084 | NA | NA | 300 | 1 000 |
| 332 | *Cycas_aenigma* | DD | 12.87560451 | NA | NA | NA | NA |
| 333 | *Cycas_indicaA.* | DD | 33.75954797 | NA | NA | NA | NA |
| 334 | *Ceratozamia_brevifrons* | DD | 26.68981432 | NA | NA | NA | NA |
| 335 | *Encephalartos_nubimontanus* | NA | 34.26228554 | NA | NA | NA | 1 000 |
| 336 | *Encephalartos_woodii* | NA | 30.93709813 | NA | NA | NA | NA |
| 337 | *Encephalartos_brevifoliolatus* | NA | 23.34009366 | NA | NA | 1 300 | 1 500 |
| 338 | *Encephalartos_relictus* | NA | 12.5674151 | NA | NA | 400 | 600 |
| 339 | *Zamia_lindleyi* | DD | 19.07345031 | NA | NA | NA | NA |
